# A Time–Frequency Domain Diagnosis Network for ICE Fault Detection

**DOI:** 10.3390/s25237139

**Published:** 2025-11-22

**Authors:** Daijie Tang, Zhiyong Yin, Demu Wu, Hongya Qian

**Affiliations:** China Ship Scientific Research Center, Wuxi 214082, China; yinzy@cssrc.com.cn (Z.Y.); wudemu@cssrc.com.cn (D.W.); qianhongya@cssrc.com.cn (H.Q.)

**Keywords:** internal combustion engines, deep learning, fault diagnosis, condition monitoring, CNN, ResNet

## Abstract

Internal combustion engines (ICEs) are prone to faults such as abnormal injection pressure and valve clearance, but traditional diagnosis methods struggle with feature extraction and require large data volumes, limiting real-time applications. Deep learning approaches like CNN and LSTM have improved accuracy but often fail to capture both time and frequency domain features efficiently. This study proposes a Time–Frequency Domain Diagnosis Network (TFDN) that integrates a time-domain path (using residual networks and self-attention mechanisms for sequential feature extraction) and a frequency-domain path (using CNNs for spatial feature extraction). The model employs Swish activation functions and batch normalization to enhance training efficiency. Validated on a six-cylinder diesel engine with 12 fault types, TFDN achieved an accuracy of 98.12%~99.79% in full-load conditions, outperforming baselines like CNN, ResNet, and LSTM. Under mixed operating conditions, TFDN maintained high accuracy, precision, and recall, and demonstrated robustness with limited data (60%~70% accuracy at 5 samples per fault). TFDN effectively combines time-frequency features to improve diagnostic accuracy and stability, enabling real-time fault detection with reduced data dependency. It offers a practical solution for ICE condition monitoring.

## 1. Introduction

Internal combustion engines (ICEs) serve as one of the core power sources for mechanical equipment. Due to their high thermal efficiency and convenient mobility, they are currently widely applied in transportation, construction machinery, power generation units, and general aviation sectors [1]. Increasingly strict environmental protection requirements have led to the growing complexity of ICEs structures, resulting in a gradual increase in the probability of malfunctions [2]. As the primary power source for most mechanical equipment, any failure in an ICE could lead to economic losses or even accidents. Monitoring the abnormal operating conditions of ICEs is of significant importance for enhancing safety and preventing the occurrence of failures [3].

Abnormal condition monitoring requires early identification of potential issues. Operational parameters of ICEs, such as rotational speed, water temperature, oil pressure, or indirect signals like vibration and noise, can all serve as signal sources for identifying abnormal states [4,5,6]. Among these, vibration signals are the most widely used due to their rich information content, simplicity of measurement, and high signal-to-noise ratio. Many researchers decompose and extract vibration signals to obtain fault-sensitive components and monitor fault occurrences based on thresholds established under normal operating conditions. For instance, Chegini et al. employed Ensemble Empirical Mode Decomposition combined with Wavelet Packet Decomposition to extract frequency-domain-sensitive signals from bearing vibrations, effectively detecting the onset of degradation [7]. Han et al. proposed using Local Mean Decomposition to extract multiple components of bearing faults and then utilized dynamic information entropy as a feature for fault recognition [8]. Xu et al. utilized Variational Mode Decomposition to extract bearing fault characteristics and simultaneously applied a multi-point kurtosis deconvolution method to enhance the impact components [9]. While these methods can effectively extract fault-sensitive features and contribute to fault mechanism research, they require strong expert knowledge and involve complex data processing workflows, typically allowing only offline analysis and not enabling online recognition.

With the development of deep learning, the accuracy of fault diagnosis has gradually improved, and online abnormal condition monitoring for ICEs is also becoming feasible. Hasan et al. converted multi-sensor fault signals into images using STFT and then fused and input them into a CNN (Convolutional Neural Network) for identification [10]. However, the process of converting signals into images is unnecessary and time-consuming, making it difficult for real-time diagnosis. Liu et al. designed a multi-task CNN model for speed and load identification, improving fault recognition accuracy [11]. CNNs can only capture spatial characteristics of the signal, failing to capture temporal features or establish long-term dependencies. As a result, RNNs (Recurrent Neural Networks) and attention mechanism networks emerged. Zhang et al. applied a Bidirectional RNN model for fault detection in chemical processes, achieving good results [12]. Shiney et al. developed a gasket inspection system using a multi-layer CNN model, which ensures the correct alignment of the radiator gasket through image recognition. The diagnostic results of the actual dataset demonstrate the effectiveness of the system [13]. Soheil et al. utilized deep learning-based image segmentation techniques to achieve fault diagnosis of solenoid starters in DC motors [14]. Currently, most researchers utilize deep learning models for fault diagnosis by converting vibration signals into images or similar approaches for feature extraction. However, one-dimensional signals not only contain time-domain information but also frequency domain characteristics, and both types of information are crucial for fault-related analysis.

To address the challenges of complex feature extraction and high data dependency in current internal combustion engine fault diagnosis, this paper aims to develop a deep learning model that integrates time-domain and frequency-domain information for comprehensive feature representation. The proposed Time–Frequency Domain Diagnosis Network (TFDN) leverages residual structures and self-attention mechanisms to capture temporal dependencies, while utilizing CNNs for spectral features, enhancing diagnostic accuracy and robustness. Through experimental validation on simulated diesel engine faults, we demonstrate that TFDN achieves superior performance compared to state-of-the-art methods, maintaining high accuracy even under data-scarce conditions. The main conclusion is that TFDN provides an efficient, end-to-end solution for real-time fault detection, facilitating reliable monitoring of ICE health.

## 2. Methods

### 2.1. Residual Network Structure

**Figure 1:**
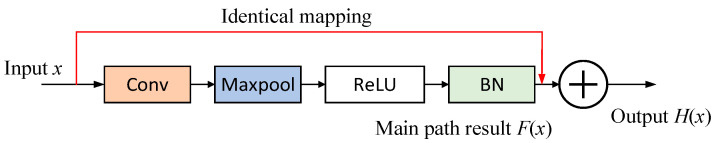
ResNet structure.

The residual structure was designed to address the degradation caused by deepening convolutional layers [15]. By incorporating an “identity mapping” structure, it preserves the original information from each layer, thereby mitigating the issue of vanishing gradients. The residual structure is illustrated in Figure 1. Additionally, since the “identity mapping” is implemented through addition operations, it does not introduce extra parameters. Compared to stacked structures, residual structures can increase network depth without increasing computational complexity. The calculation formula for the residual structure is as follows:

(1) $$H_{n}(x_{j})=F_{n}(x_{j})+W_{n}x_{j}$$

In the *n*-th residual structure, $F_{n}(x)$ represents the output of the internal structure within the residual block, $x_{j}$ denotes the *j*-th input data batch, *W_n_* is the dimension conversion matrix, and $H_{n}\left(x_{j}\right)$ represents the output of the *n*-th residual structure. The introduction of the residual structure makes it possible to construct deeper network architectures while improving network performance. This is because the network learns the differential information between the input and the desired output, enhancing its ability to capture complex patterns and dependencies in the data.

The main path of the residual network includes convolutional layers, pooling layers, activation functions, and batch normalization layers. Given that vibration signals are all 1D signals, the calculation formula for 1D convolution is as follows:

(2) $$y_{k}^{l}=\omega_{k}^{l}x_{j}^{l}+b_{k}^{l}$$

In the formula, $y_{k}^{l}$ represents the result obtained from the *k*-th convolutional kernel in the *l*-th convolutional layer. $\omega_{k}^{l}$ and $b_{k}^{l}$ represent the weights and bias of the *k*-th convolutional kernel in the *l*-th layer. $x_{j}^{l}$ represents the *j*-th input batch in the *l*-th convolutional layer.

The Batch Normalization Layer (BN) [16] primarily performs normalization on the input layer data and its normalization calculation is performed per channel. The calculation formula for BN is as follows:

(3) $$y=\frac{x-\mu }{\sqrt{\sigma^{2}+\varepsilon }}\gamma +\beta$$

In this context, *y* and *x* represent the output and input, respectively. μ and σ^2^ denote the mean and variance of each feature map in the current batch. ε is a small constant added to the denominator to avoid division by zero. *γ* and *β* are learnable parameters.

### 2.2. The Self-Attention Mechanism

Vaswani et al. published “Attention Is All You Need” in 2017 [17] and introduced the Transformer model, whose core component is the self-attention mechanism (SAM). SAM significantly enhances the ability to model relationships between different positions within the input sequence, establishing mutual connections within the sequence. Compared to traditional RNNs, SAM can better capture global information and address long-range dependency issues, while outperforming CNN methods in handling sequence-based tasks.

The computational principle of the self-attention mechanism is illustrated in Equations (4)–(7), where *Q*, *K*, and *V* represent the query vector, key vector, and value vector, respectively. These vectors are obtained by performing matrix multiplication with the input signal.

(4) $$Q=W_{Q}x$$

(5) $$K=W_{K}x$$

(6) $$V=W_{V}x$$

(7) $$Attention(Q,K,V)=soft\max(\frac{QK^{T}}{\sqrt{d_{K}}})V$$

In this context, *W_Q_*, *W_K_*, and *W_V_* represent weight matrices, which are multiplied with the input signal to obtain the query matrix *Q*, key matrix *K*, and value matrix *V*, respectively. Here, *d_K_* denotes the dimension of the *Q* matrix. By transposing and multiplying the *Q* and *K* matrices, each query vector *q_i_* at position *i* is connected to all key vectors *k_j_* across positions, resulting in an attention score matrix *A*. This matrix is then normalized using softmax to obtain the attention weight matrix *W*. Finally, *W* is multiplied with the value matrix *V*, which represents the sequence’s feature information, to produce the output.

### 2.3. Swish Activation Function

In deep learning, the choice of activation function has a significant impact on training efficiency and accuracy. The most successful activation function to date is the rectified linear unit (ReLU). Due to its simple principle and high computational efficiency, it has been widely adopted. However, ReLU suffers from the “dead ReLU” problem for negative input values. In 2017, Prajit et al. [18]. utilized a method combining automated search with reinforcement learning to discover new activation functions. They found that the Swish function performs better than ReLU for deep networks and improves accuracy for most existing models while maintaining a simple principle. The Swish function is expressed as shown in Equation (8):

(8) $$\mathrm{Swish}(x)=x\mathrm{sigmoid}(\beta_{t}x)=\frac{x}{1+e^{-\beta_{t}x}},\;\beta_{t}=0.1,\;1,\;10$$

**Figure 2:**
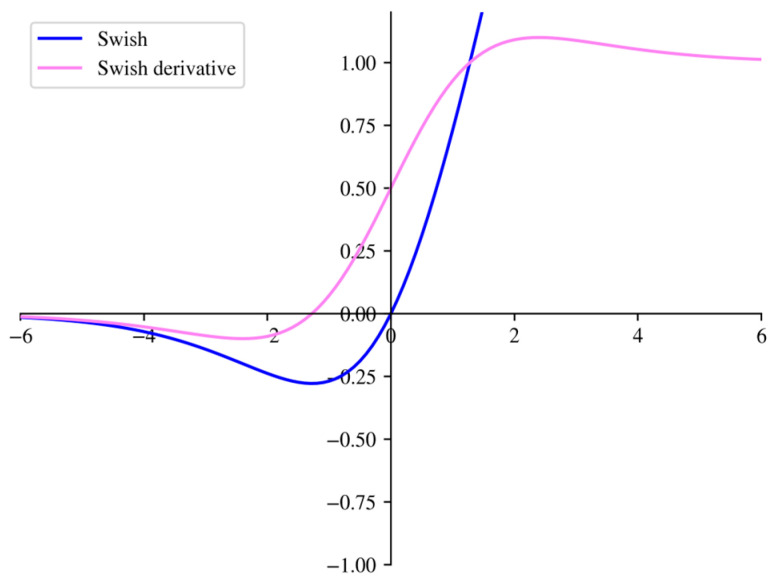
Swish function and its derivatives.

In the equation, *β*_t_ is an adjustable parameter, and the authors recommend possible values of 0.1, 1, and 10. Figure 2 illustrates the function and derivative function images of the Swish function when *β_t_* =1. As can be seen, the Swish function exhibits a linear-like nature similar to ReLU when the input is positive. However, it retains activation effects even when the input is negative. Additionally, unlike the ReLU function and its derivative, which exhibit abrupt changes at zero, both the derivative of Swish and its derivative function are relatively smooth.

## 3. Time–Frequency Domain Diagnosis Network

Internal combustion engines are reciprocating machines, and their vibration signals primarily originate from periodic combustion explosion impacts and the knocking caused by the reciprocating motion of the piston. Additionally, vibrations are induced by the operation of various components, coupled with the mutual coupling of vibrations during transmission. As a result, the measured signals often contain a large amount of complex information. Time domain vibration signals can provide information about the temporal variations in vibrations, including their amplitudes, waveform characteristics, and periodicity, among others. When reflected in the frequency spectrum, vibration signals manifest as energy at different frequencies and their distribution characteristics. Conventional CNN or CNN-Transformer models commonly employ one of three strategies: converting temporal signals into images, or utilizing solely temporal-domain or frequency-domain data as the input for fault diagnosis. However, when a fault occurs, it may be characterized by changes in the time-domain waveform or alterations in the vibration energy and distribution in the frequency domain. While employing deeper networks and ample data can enhance diagnostic accuracy, a more efficient and direct approach is to design dedicated networks for time-domain and frequency-domain feature extraction. Combining time-domain and frequency-domain signals provides a more comprehensive diagnostic information, which helps to improve the accuracy. For diagnostic models, simultaneously incorporating time-domain and frequency-domain information improves the model’s feature representation capability, enabling complementary features.

Based on the above analysis, this section will establish a deep learning model that integrates both time domain and frequency domain signals from vibration signals. Assume the input signal is $x\in {\mathbb{R}}^{B\times 1\times L}$, where *B* denotes the batch size and *L* represents the signal length. The overall forward propagation formula is as follows. The FFT(x) denotes the Fourier transform, and “[,]” represents the concatenation operation.

(9) y=Classifier([TimeDomain(x),FreqDomain(FFT(x))])

In the time domain feature extraction branch, the single-head self-attention mechanism (SAM) effectively extracts sequential features by modeling within sequences to capture global information and long-range dependencies. However, due to the large number of parameters in SAM, directly extracting features from raw signals is challenging. Therefore, multiple convolutional and residual convolutional network layers are set before the SAM layer to initially extract signal features, ultimately forming a time-domain feature extraction network. By using traversal methods, we have sought to reduce the number of residual blocks and convolutional layers to minimize the model parameters. By adding BN and replacing the activation function with the Swish function, the performance of the time-domain feature extraction model is gradually improved. The network architecture is detailed in Table 1. In the table, “64@3×1” indicates 64 output channels and a convolution kernel width of 3; P denotes the dropout rate; FC stands for a fully connected layer; and c represents the number of classes.

The frequency domain signal is characterized by a line spectrum, which essentially contains only spatial distribution features. Since CNNs have strong capabilities in extracting spatial features, they are suitable for frequency-domain feature extraction. Through testing, the performance of the multi-layer convolutional neural network is already sufficient to extract the frequency-domain features of the signal. Therefore, for frequency-domain signals, a set of CNN networks is utilized for feature extraction, including three layers of convolutional layers, pooling layers, and batch normalization layers, with the Swish function used for activation. The frequency domain feature extraction network is shown in Table 2. Among them, the parameters of the FFT layer are not learnable.

**Figure 3:**
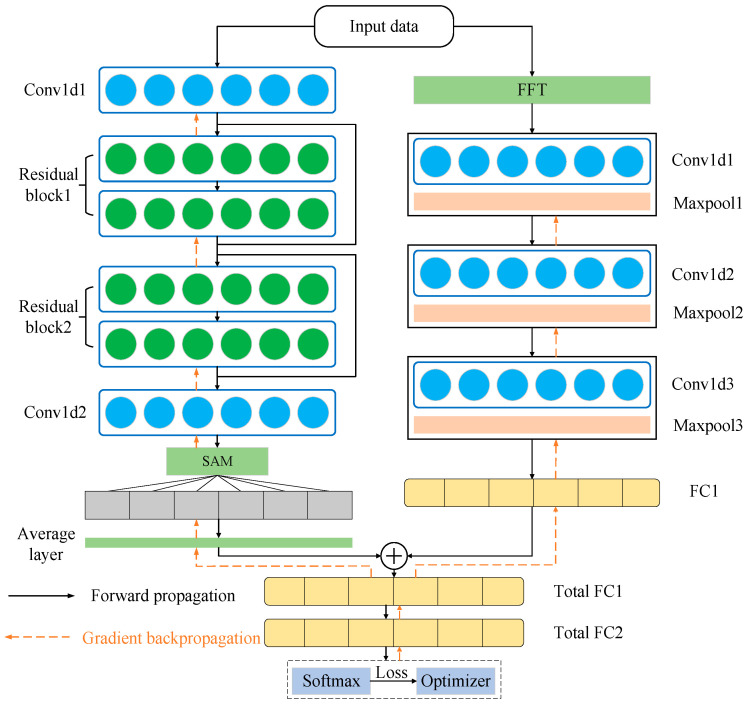
Time–frequency dual-line diagnostic network structure.

By combining the time domain and frequency domain feature extraction networks, we can obtain the feature set of the signal in both time and frequency domains. After merging the final feature layers and performing classification, the fault diagnosis results can be obtained. The last two layers of the networks in Table 2 and Table 3 are shared. Figure 3 illustrates a schematic diagram of the Time–Frequency Domain Diagnosis Network (TFDN). Based on the above partial content, the complete forward propagation expression can be represented by Equations (10)–(12).

(10) $$y_{time}=\frac{1}{L}\sum_{i=1}^{L}\mathrm{Attention}{\left(\mathrm{ResNet}(x)\right)}_{:,:,i}$$

(11) $$y_{freq}=W_{f}\cdot \mathrm{flatten}\left(\mathrm{FreqCNN}\left(\left|\mathrm{FFT}{\left(x\right)}_{:1024}\right|\right)\right)$$

(12) $$y=W_{3}\cdot \mathrm{Swish}\left(\mathrm{BN}\left(W_{2}\cdot \mathrm{Dropout}\left(W_{1}\cdot \left[y_{time},y_{freq}\right]+b_{1}\right)\right)+b_{2}\right)+b_{3}$$

where $y_{time}$ represents the extracted time domain features, and $y_{freq}$ represents the extracted frequency domain features. *W* denotes the weight matrix of the neural network, and *b* denotes the bias matrix.

## 4. Case Study

**Figure 4:**
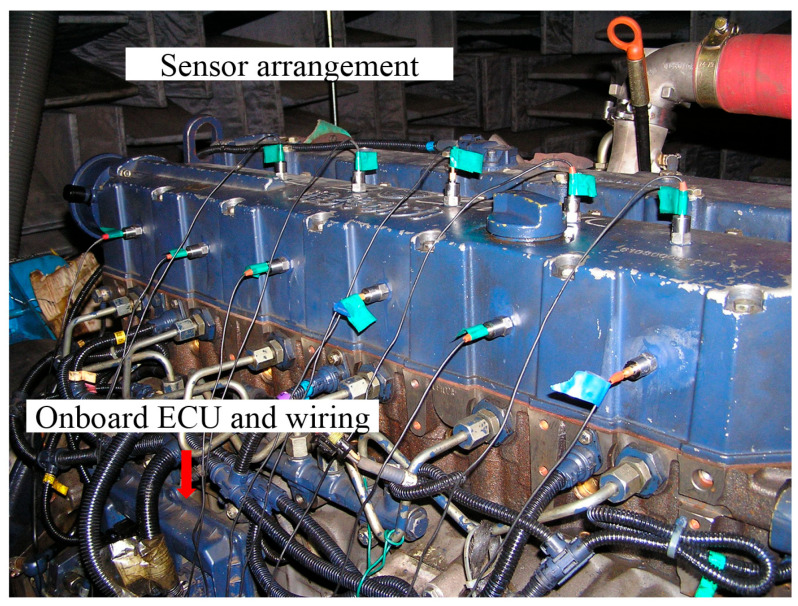
Sensor layout for bench experiment.

**Figure 5:**
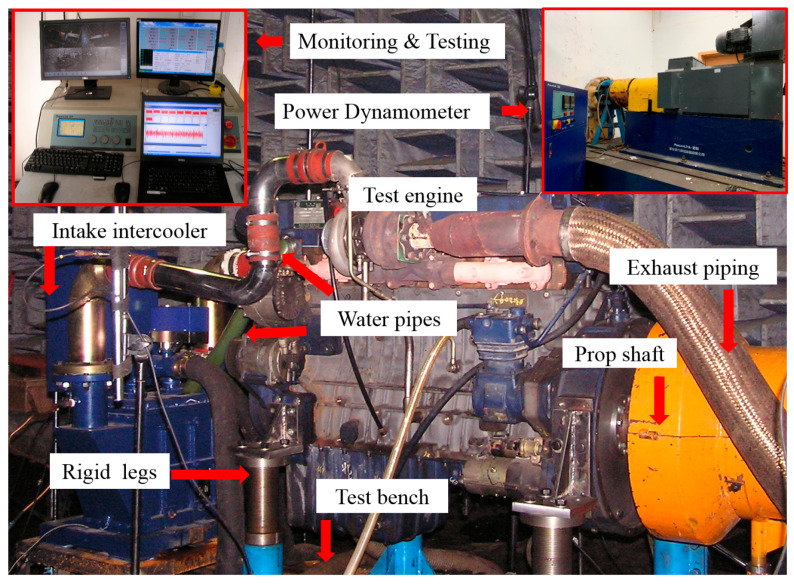
Display of diesel engine and dynamometer.

To validate the effectiveness of the ICE abnormal state identification method, we designed a simulated fault test on an inline six-cylinder diesel engine and collected vibration acceleration data from the cylinder head and engine block, as shown in Figure 4. The diesel engine test bench system consists of two parts: the test bench and the signal acquisition system. The test bench includes the diesel engine, an electric dynamometer, a driveshaft, as well as water, oil, and air piping systems, as shown in Figure 5. The signal acquisition system comprises sensors, wiring harnesses, data acquisition frontends, and a test computer. Vibration acceleration was measured using PCB’s 621B40 type sensor (Depew, NY, USA), and engine speed was tested using the SPSE-115 type photoelectric sensor from MONARCH Company (Medicine Hat, AB, Canada).

Nahim et al. conducted a review and analysis of the main fault types in diesel engines and statistically analyzed the probabilities of various types of faults [19]. The results showed that faults in the fuel injection and fuel supply systems, waterway leakage faults, and valve seat faults have the highest probabilities of occurrence. Waterway leakage, which affects engine cooling, can be easily detected using temperature sensors. Faults in the fuel supply system and valve seat system involve complex mechanical structures, making diagnosis more challenging. For these two types of faults, we designed four categories of faults: abnormal injection pressure, abnormal fuel injection, abnormal injection advance angle, and abnormal valve clearance, including different degrees of abnormal states. The specific faults and their parameters are shown in Table 3, comprising a total of twelve distinct states.

During the test, the sampling frequency was set to 25,600 Hz, and the test speeds included three levels: 1600 r/min, 2000 r/min, and 2300 r/min. The engine operated under two load conditions: 50% load and 100% load. Therefore, when intercepting data, the length was determined based on the duration of a single combustion cycle at the minimum speed (0.075 s), so *L* should be greater than 1920. In this paper, *L* was set to 2048. When intercepting data, a 20% overlap sampling ratio is adopted to increase the data volume. After data interception, no windowing, filtering, or normalization operations were performed.

The proposed method is compared with Random Forest (RF), Long Short-Term Memory (LSTM), CNN, and ResNet18. The parameter settings for each method are shown in Table 4. Among these, the time-domain extraction network (TDEN) represents the time-domain feature extraction part of the proposed method in this paper. The parameter counts of each model used are listed in Table 4, where the parameters of RF are calculated based on an average of 1500 nodes per decision tree. It can be seen that the number of parameters of the proposed model is smaller than that of ResNet18 and LSTM, but larger than that of the CNN and RF model.

Using the methods listed in Table 4 to diagnose engine fault data, we select data from three sensors: the first cylinder head (1H), the first cylinder block (1B), and the third cylinder head (3H). Each dataset includes three engine speeds: 1600 rpm, 2000 rpm, and 2300 rpm. Among the fault types, abnormal injection pressure, abnormal fuel injection, and abnormal injection advance angle occur simultaneously in all cylinders, while abnormal valve clearance occurs only in the first cylinder. Each fault type has 200 samples, with each sample containing data for one engine cycle. The ratio of the training set, test set, and validation set is 3:1:1., and each model was trained for 200 epochs.

The diagnostic results under full load and single-speed conditions are shown in Table 5. The results indicate that RF lacks sufficient feature extraction capability, achieving only 20% accuracy, as RF heavily relies on preprocessing and cannot perform end-to-end diagnosis. LSTM also shows low diagnostic accuracy, as this method performs well for regression tasks but is less effective for classification tasks. CNN has limited temporal feature extraction capability, and due to the complexity of engine time-domain signals, its accuracy fluctuates between 50% and 85%. ResNet, with its deeper network layers, demonstrates excellent feature extraction performance, achieving an accuracy rate of 88.75%~99.58%, significantly outperforming CNN. The TFEN method has fewer network layers than ResNet, but its accuracy is very close to that of ResNet, suggesting that the introduction of SAM effectively enhances temporal feature extraction capability. The TFDN method achieves an accuracy of 98.12%~99.79%, indicating that the introduction of frequency domain features significantly improves diagnostic stability.

To verify the diagnostic performance of the algorithm on mixed speed and load operating conditions data, datasets from sensors 1H, 1B, and 3H were combined and shuffled. These datasets included speeds of 1600 r/min, 2000 r/min, and 2300 r/min, as well as loads of 50% and 100%, with each condition containing 200 samples. The ratio of the training set, test set, and validation set is 3:1:1. To ensure a uniform dimension, each sample was truncated to 2048 data points. The diagnostic results obtained using the aforementioned algorithms, including accuracy, precision, and recall, are shown in Table 6. As the amount of training data increased, the accuracy of LSTM improved. However, the accuracy of ResNet, TDEN, and TFDN slightly decreased. Nevertheless, TFDN still demonstrated the best performance.

**Figure 6:**
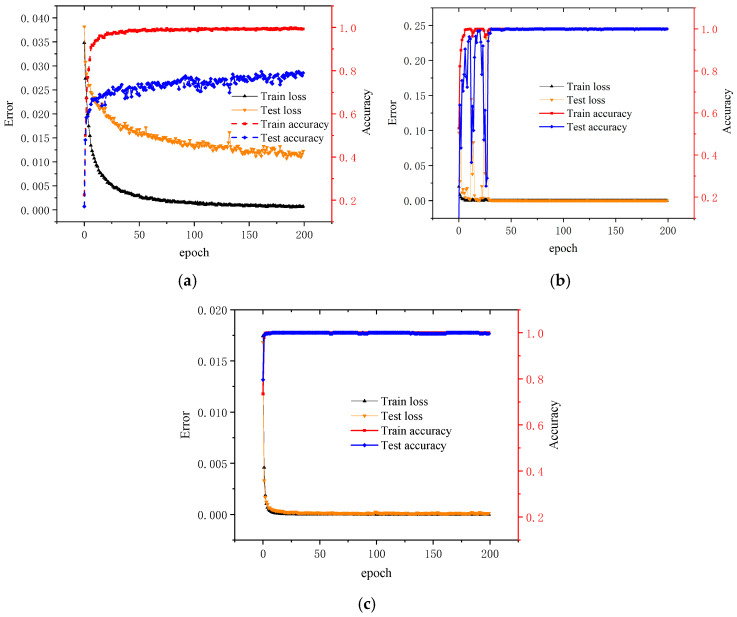
Accuracy and loss curves of each algorithm, (**a**) CNN model (**b**) ResNet model (**c**) TFDN model.

By observing the changes in error and accuracy during the training process, a better assessment of model performance can be made. Based on the results in Table 6, CNN, ResNet, and TFDN methods were selected for comparison, with the 3H dataset as an example. Figure 6a–c show the loss and accuracy changes during the training process of the aforementioned three algorithms. The results indicate that the CNN has average fitting capability and insufficient time-domain feature extraction ability. Although ResNet achieves better fitting in the end, there are larger fluctuations in the first 30 epochs due to its deep model architecture, which requires a higher data quantity and training time. The proposed TFDN model demonstrates fast convergence, the best fitting performance, and the highest training efficiency.

**Figure 7:**
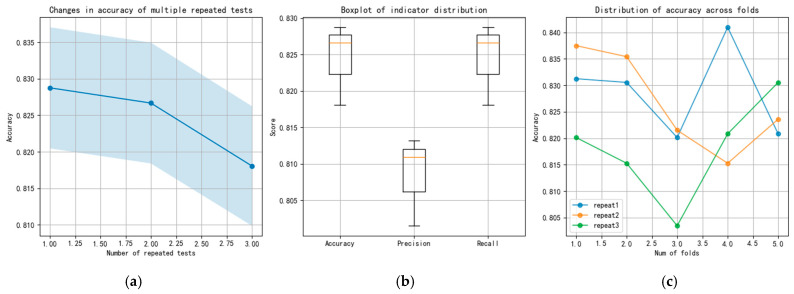
Results of repeated experiments and cross-validation for the CNN; (**a**) changes in accuracy of repeated tests; (**b**) boxplot of indicator; (**c**) distribution of accuracy across folds.

**Figure 8:**
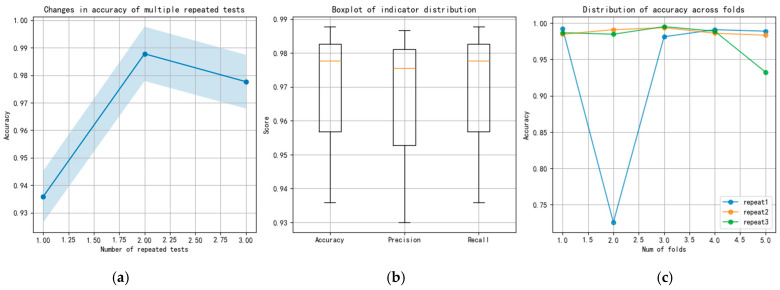
Results of repeated experiments and cross-validation for the ResNet; (**a**) changes in accuracy of repeated tests; (**b**) boxplot of indicator; (**c**) distribution of accuracy across folds.

**Figure 9:**
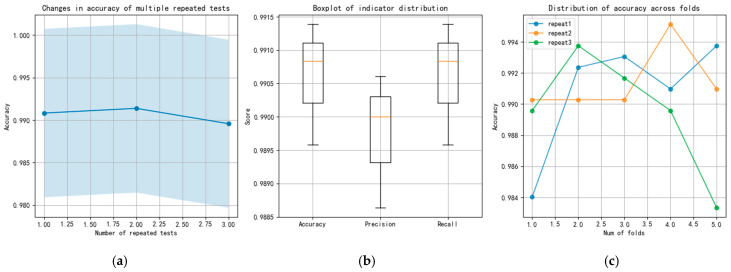
Results of repeated experiments and cross-validation for the TFDN; (**a**) changes in accuracy of repeated tests; (**b**) boxplot of indicator; (**c**) distribution of accuracy across folds.

To compare the stability of various methods during multiple calculations, the performance of the CNN, ResNet, and TFDN methods in three repeated five-fold cross-validation tests is compared below. Taking the combined dataset of all rotational speeds and all loads from sensor 3H as an example. The changes in the accuracy, precision, and recall indicators of each method are also presented. The results of the three methods are shown in Figure 7, Figure 8 and Figure 9, respectively. The blue shadows in Figure 7a, Figure 8a, and Figure 9a represent the overall error range of each method across three repeated tests. Figure 7 shows that in the repeated tests of the CNN method, all indicators vary between 0.8 and 0.85, with moderate accuracy. Both ResNet and TFDN achieve relatively high accuracy. Among them, the accuracy of ResNet fluctuates between 0.93 and 0.99, but it is not very stable—its accuracy drops below 0.75 in one of the folds. In the repeated tests, the accuracy of the TFDN method fluctuates around 0.99, with the lowest accuracy near 0.984. It is the best performing among the three methods. As can be seen from the boxplot, among the three methods, the indicators of CNN and TFDN are relatively stable, while those of ResNet show significant fluctuations.

**Figure 10:**
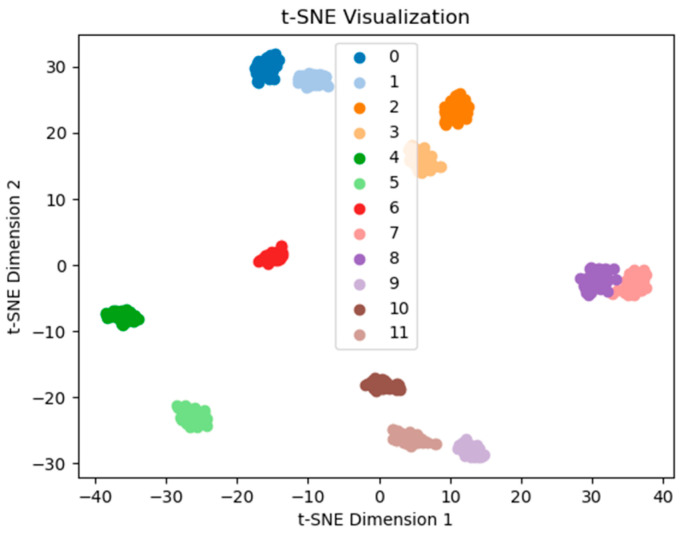
TFDN feature extraction visualization.

**Figure 11:**
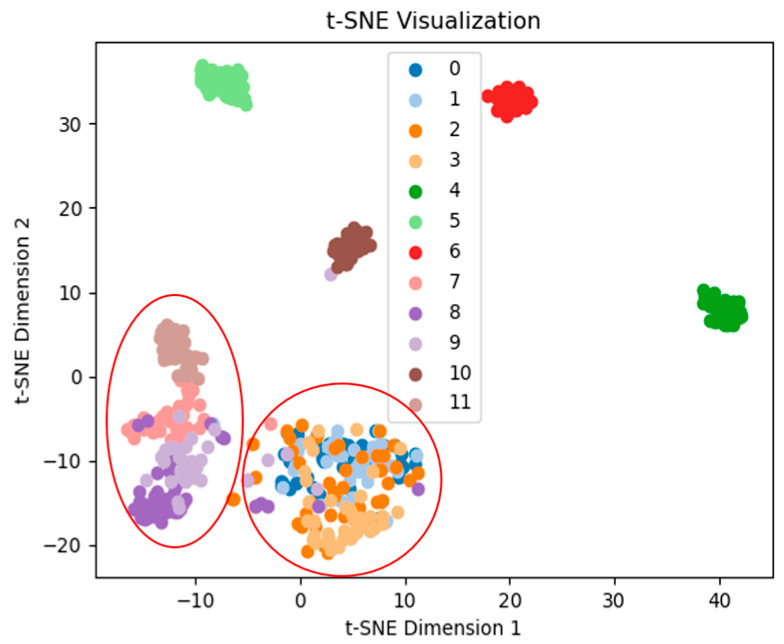
CNN feature extraction visualization.

**Figure 12:**
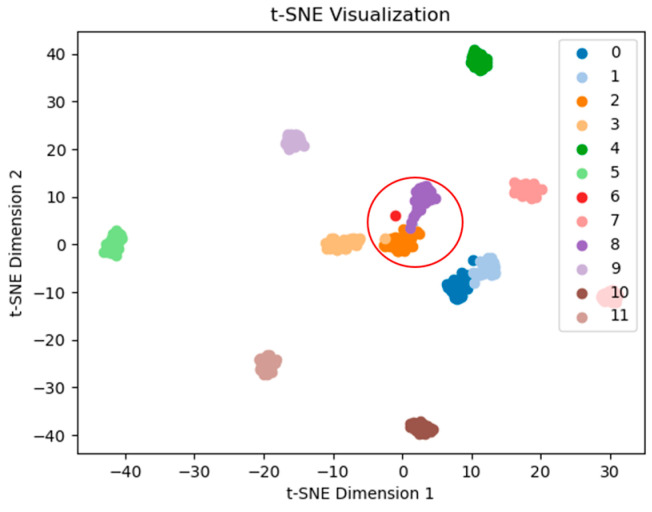
ResNet feature extraction visualization.

The feature extraction results of the network are visualized using t-SNE, with the 3H dataset at 2000 r/min as an example. The feature extraction performance of the network is demonstrated before the final classification. Figure 10 shows that the proposed TFDN has a good clustering effect, with clear separation boundaries between various types of data. Next is the ResNet method, which only has confusion between two types of fault data (see Figure 11). Figure 12 shows that the clustering effect of CNN after feature extraction is poor, with confusion among multiple types of fault data. To quantify the t-SNE results, we calculated three parameters for each result: Silhouette Score, Calinski–Harabasz index, and Davies–Bouldin index. Among them, the larger the Calinski–Harabasz index, the better; the smaller the other two indices, the better. The results are shown in Table 7. It can be seen that the proposed method performs well in all three indices, and ResNet also achieves good results.

Data scarcity is a common issue in real-world scenarios, especially for fault data, which is typically scarce and valuable. It is necessary to study the diagnostic capability of fault diagnosis models under conditions of insufficient training data. Therefore, in the next step, the accuracy changes in various methods under conditions of missing training data will be investigated, using the 3H 2000 r/min dataset as an example. The test ratio of the dataset will be modified, allowing the test ratio to vary between 0.2 and 0.975. Originally, each fault type contains 200 data samples; therefore, when the test rate is 0.975, each category of data contains only 5 training samples.

**Figure 13:**
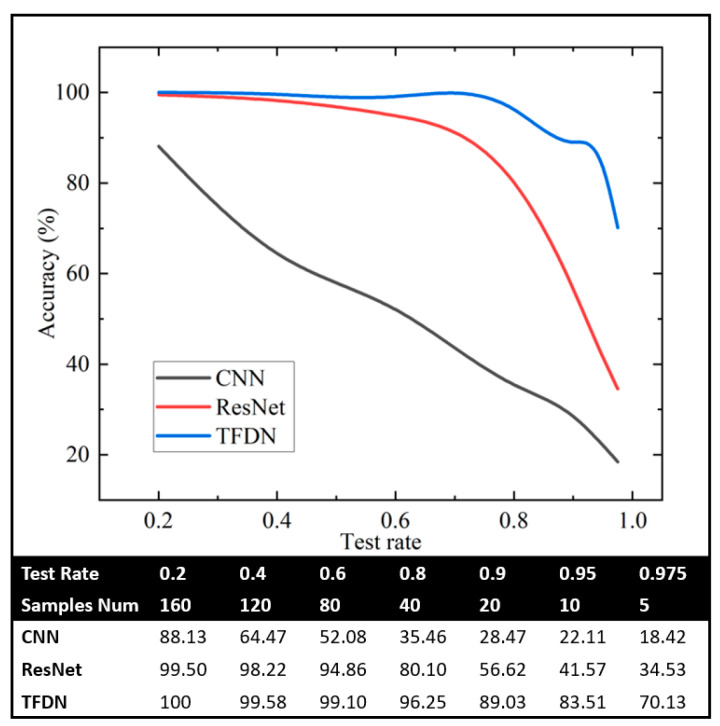
Variation in algorithm accuracy for different test ratio cases.

**Figure 14:**
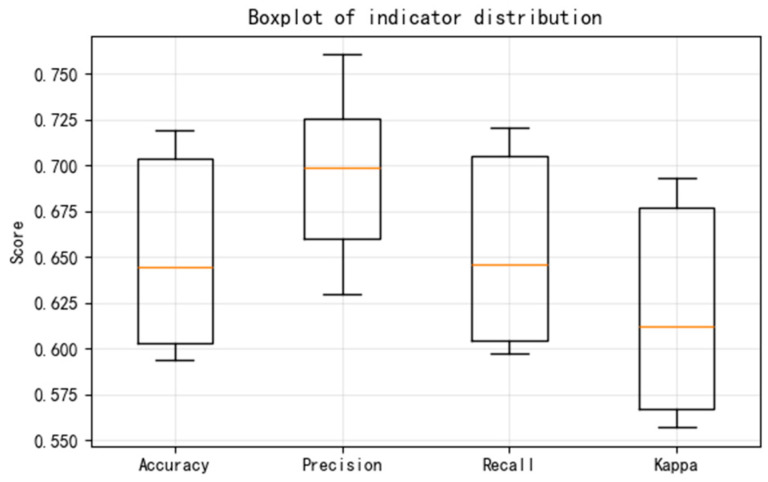
Boxplot of indicators for TFDN repeated tests (Test rate = 0.975).

The diagnostic results are shown in Figure 13. As the test rate increases, the amount of training data decreases, leading to a continuous decline in the diagnostic accuracy of all three algorithms. The accuracy of CNN drops rapidly with the increase in test rate, and when the test rate is 0.975, its accuracy is only 18.42%. For ResNet, the accuracy does not decrease significantly until the test rate exceeds 0.6. When the test rate is 0.975, its accuracy is 34.53%. On the other hand, TFDN’s accuracy begins to drop rapidly only when the test rate exceeds 0.9. Even when each category has only five training samples, TFDN still achieves an accuracy of 70%. To verify the stability of TFDN under the condition of data scarcity, the proposed method is subjected to 5 repeated tests, and the variation ranges of various indicators are calculated. The results are shown in Figure 14. Figure 14 indicates that in multiple tests, the proposed method achieved an average accuracy of 65.26% (with a minimum of 60.36% and a maximum of 71.88%), an average precision of 70.48%, and an average recall of 65.46%. This demonstrates that the method can maintain a certain diagnostic effect even when data is scarce. The results demonstrate that the proposed TFDN model maintains a high level of accuracy even when data is scarce, indicating that the algorithm has a certain capability to handle the risk of data deficiency.

## 5. Conclusions

The proposed TFDN framework integrates independent and complementary time domain and frequency domain feature extraction pathways, providing an approach to address key limitations of existing internal combustion engine (ICE) fault diagnosis methods. The time domain pathway adopts residual blocks and self-attention mechanisms (SAM) to model complex temporal dynamics and long-range dependencies inherent in reciprocating engine vibrations, while the frequency domain CNN pathway is used to extract spatial distribution patterns of spectral energy.

Validation experiments were conducted on a purpose-built inline six-cylinder diesel engine test bench, covering 12 fault conditions including abnormal injection pressure, abnormal fuel injection, abnormal injection advance angle, and abnormal valve clearance. Experimental results show that the TFDN has certain diagnostic accuracy and robustness, and its performance is better than that of comparison models such as Random Forest (RF), Long Short-Term Memory (LSTM), standard CNN, ResNet18, and the time domain-only component (TDEN) of TFDN. Among them, the fusion of frequency domain features plays an important role in improving diagnostic stability, especially under variable operating conditions.

The scarcity of labeled fault data is one of the main challenges in practical fault diagnosis. TFDN exhibits resilience in low-data scenarios. As Figure 9 illustrates, when the training data per fault class was drastically reduced to only 5 samples (test ratio = 0.975), TFDN maintained a high accuracy of 70%, which is higher than that of CNN (about 18%) and ResNet18 (about 35%) under the same conditions. This ability to learn from limited data is presumably related to the feature representation obtained by the dual-path architecture and regularization mechanisms such as dropout layers and batch normalization in the design, which endows it with application potential in practical scenarios where fault data acquisition is difficult or costly.

This study explores the issues of complex feature extraction and high data dependency in current deep learning-based ICE diagnosis. The TFDN model presents feasible solution for ICE condition monitoring systems. Its ability to perform end-to-end learning directly from raw 1D signals eliminates the need for computationally expensive signal pre-processing or transformation (e.g., to images via STFT), enhancing its suitability for deployment. However, the method proposed is based on deep neural networks and is a black-box approach with poor interpretability, which cannot facilitate the research on ICE fault mechanisms. Meanwhile, the introduction of the attention mechanism will significantly increase the hardware computational load, which is not conducive to online diagnosis. At present, many scholars have introduced physical information into deep learning models to improve the interpretability and stability of the models [20,21], which is also a future research direction.

In addition, the data in the paper are derived from simulated faults in the laboratory; in the real world, fault data contain greater noise, and the difficulty of diagnosis will also increase significantly. At this time, it may be necessary to conduct research on transfer learning methods. As reported in Refs. [22,23], there should be a clear mapping method for the mapping relationship between the source domain of laboratory fault data and the target domain of actual operating data.

Future work will focus on (a) further optimizing the network architecture for computational efficiency and real-time inference on embedded systems; (b) validating the model’s generalizability across a wider range of engine types, fault modes, and noise conditions; (c) exploring online learning strategies to adapt the model to engine aging and varying operational environments; and (d) investigating the fusion of vibration data with other sensor modalities (e.g., acoustic, thermal, pressure) within the TFDN framework for even more robust and comprehensive diagnostics.

## Figures and Tables

**Table 1:** Time domain feature extraction network.

Layer Number	Network Layer	Parameters	Output Size
1	Conv1d	3 × 1	(batch, 64, L)
2	BN	-	(batch, 64, L)
3	Swish	-	(batch, 64, L)
4	ResidualBlock	Conv1d 64@3×1 BN Swish Conv1d 64@3×1 BN	(batch, 64, L)
5	ResidualBlock	Conv1d 64@3×1 BN Swish Conv1d 64@3×1 BN	(batch, 64, L)
6	Conv1d	3 × 1	(batch, 128, L)
7	BN	-	(batch, 128, L)
9	SAM	Single-head	(batch, 128, L)

**Table 2:** Frequency domain feature extraction network.

Layer Number	Network Layer	Parameters	Output Size
1	FFT layer	single-sided spectrum	(batch, 1, 1024)
2	Conv1d	3 × 1	(batch, 16, 1024)
3	MaxPool1d	2 × 1	(batch, 16, 512)
4	BN	-	(batch, 16, 512)
5	Swish	-	(batch, 16, 512)
6	Conv1d	3 × 1	(batch, 32, 512)
7	MaxPool1d	2 × 1	(batch, 32, 256)
8	Swish	-	(batch, 32, 256)
9	Conv1d	3 × 1	(batch, 16, 256)
10	MaxPool1d	2 × 1	(batch, 16, 128)
11	BN	-	(batch, 16, 128)
12	Swish	-	(batch, 16, 128)
13	Flatten	-	(batch, 2048)

**Table 3:** Abnormal ICE condition setting.

Fault Type	Parameters	Marks
Abnormal injection advance angle	−1 °CA	I
	−2 °CA	II
	+1 °CA	III
	+2 °CA	IV
Abnormal fuel injection	−75%	V
	−25%	VI
Normal	—	VII
Abnormal injection pressure	−200 bar	VIII
	+200 bar	IX
Abnormal valve clearance	−0.05 mm	X
	+0.05 mm	XI
	+0.10 mm	XII

**Table 4:** The parameter settings of other methods.

Methods	Parameters	Number of Parameters
RF	100 decision trees	~150,000
LSTM	Four layers of BiLSTM with 64, 64, 128, 128 neurons.	3,419,662
CNN	A four-layer Conv1d-Maxpool-BN structure, the parameters of each layer are 16@3×1, 64@3×1, 128@3×1, 32@3×1.	84,714
ResNet18	See paper [15], converting 2d networks to 1d.	3,853,068
TDEN	The proposed method without frequency feature extraction.	95,904
TFDN	The proposed method.	378,844

**Table 5:** Comparison of the diagnostic accuracy of each algorithm (%).

Sensors	Speed r/min	RF	LSTM	CNN	ResNet	TDEN	TFDN
1H	1600	16.90	8.97	71.25	99.58	98.33	99.79
	2000	16.24	10.12	74.38	96.46	93.75	98.12
	2300	15.85	16.67	71.88	98.33	97.08	99.79
1B	1600	12.54	9.23	71.67	98.96	97.29	99.58
	2000	18.75	9.41	85.00	99.58	96.88	99.79
	2300	22.10	20.11	72.29	97.50	96.25	99.17
3H	1600	17.63	9.53	69.79	99.38	94.17	99.79
	2000	15.53	10.55	57.08	88.75	85.42	99.58
	2300	18.42	14.80	68.24	96.04	93.67	99.58

**Table 6:** The results on mix condition dataset of each algorithm (%).

Sensors	Index	RF	LSTM	CNN	ResNet	TDEN	TFDN
1H	Accuracy	21.53	26.18	70.35	97.92	95.07	98.19
	Precision	22.70	28.50	70.56	97.92	95.10	98.22
	Recall	21.50	26.20	70.35	97.92	95.07	98.19
1B	Accuracy	22.91	44.65	85.97	97.78	97.50	99.79
	Precision	23.60	51.80	85.96	97.83	97.53	99.79
	Recall	22.90	44.70	85.97	97.83	97.50	99.79
3H	Accuracy	15.90	29.51	74.93	96.71	91.39	99.06
	Precision	15.40	29.90	74.64	96.41	91.45	98.97
	Recall	15.90	28.70	74.93	96.71	91.38	99.06

**Table 7:** Quantitative indicators of various methods after t-SNE dimensionality reduction.

Methods	Silhouette Score	Calinski–Harabasz Index	Davies–Bouldin Index
TFDN	0.7952	10,574.12	0.2859
CNN	0.2292	324.41	4.5948
ResNet	0.7614	6043.61	0.3274

## Data Availability

The data presented in this study are not publicly available at this time but may be obtained upon reasonable request from the authors.
